# Investigating the Mechanisms of Pollen Typhae in the Treatment of Diabetic Retinopathy Based on Network Pharmacology and Molecular Docking

**DOI:** 10.1155/2022/5728408

**Published:** 2022-01-03

**Authors:** Rongrong Zhou, De Jin, Yuqing Zhang, Liyun Duan, Yuehong Zhang, Yingying Duan, Xiaomin Kang, Fengmei Lian

**Affiliations:** ^1^Guang'anmen Hospital, China Academy of Chinese Medical Sciences, Beijing 100053, China; ^2^Beijing University of Chinese Medicine, Beijing 10029, China

## Abstract

**Objective:**

To explore the main bioactive compounds and investigate the underlying mechanism of Pollen Typhae (PT) against diabetic retinopathy (DR) by network pharmacology and molecular docking analysis.

**Methods:**

Bioactive ingredients and the target proteins of PT were obtained from TCMSP, and the related target genes were acquired from the SwissTargetPrediction database. The target genes of DR were obtained from GeneCards, TTD database, DisGeNET database, and DrugBank. The compound-target interaction network was established based on Cytoscape 3.7.2. The protein-protein interaction (PPI) network was constructed via STRING database and Cytoscape 3.7.2. Gene ontology (GO) analysis and Kyoto Encyclopedia of Genes and Genomes (KEGG) pathway enrichment analysis were visualized through DAVID database and Bioinformatics. Ingredient-gene-pathway network analysis was conducted to further screen the ingredients, target proteins, and pathways closely related to the biological mechanism on PT for DR, and molecular docking analysis was performed by SYBYL-X 2.1.1 software. Finally, the mechanism and underlying targets of PT in the treatment of DR were predicted.

**Results:**

A total of 8 compounds and 171 intersection targets were obtained based on the online network database. 7 main compounds were screened from compound-target network, and 53 targets including the top six key targets (PTGS2, AKT1, VEGFA, MAPK3, TNF, and EGFR) were further acquired from PPI analysis. The 53 key targets covered 80 signaling pathways, among which PI3K-Akt signaling pathway, focal adhesion, Rap1 signaling pathway, VEGF signaling pathway, and HIF-1 signaling pathway were closely connected with the biological mechanism involved in the alleviation of DR by PT. Ingredient-gene-pathway network shows that AKTI, EGFR, and VEGFA were core genes, kaempferol and isorhamnetin were pivotal ingredients, and VEGF signaling pathway and Rap1 signaling pathway were closely involved in anti-DR. The docking results indicated that five main compounds (arachidonic acid, isorhamnetin, quercetin, kaempferol, and (2R)-5,7-dihydroxy-2-(4-hydroxyphenyl)chroman-4-one) had good binding activity with EGFR and AKT1 targets.

**Conclusion:**

The active ingredients in PT may regulate the levels of inflammatory factors, suppress the oxidative stress, and inhibit the proliferation, migration, and invasion of retinal pericytes by acting on PTGS2, AKT1, VEGFA, MAPK3, TNF, and EGFR targets through VEGF signaling pathway, PI3K-Akt signaling pathway, Rap1 signaling pathway, and HIF-1 signaling pathway to play a therapeutic role in diabetic retinopathy.

## 1. Introduction

Diabetic retinopathy (DR), which results from chronic high blood glucose levels and almost occurs in medium and late stage of type 1 and type 2 diabetes, is one of the most serious microvascular complications of diabetes mellitus [1]. Diabetic retinopathy can result in severe visual impairment, vitreous hemorrhage, and even blindness [2]. The World Health Organization estimated that more than 93 million people worldwide are affected by DR. The rapidly rising incidence of DR is a serious threat to public health which has been put into much attention. Moreover, DR is the main cause of adult-acquired blindness [3, 4], which dramatically affects a patient's life and spirit and leads to a massive social economic burden. Currently, a variety of pharmaceutical and surgical treatments have been developed for DR [5]; however, their adverse side effects and limited therapeutic effect have contributed to making DR a challenge for clinical practice. Therefore, it is crucial to develop potential and effective therapeutic strategies to treat DR based on its pathogenesis.

Traditional Chinese medicine (TCM), as a type of alternative drug, is considered a multicomponent and multitarget treatment for diseases corresponding to complex organic targets. Thus, it is necessary to study pharmacological effects and molecular mechanisms of TCMs. Pollen Typhae (PT) is a classical and vital Chinese medicine with well-defined phytochemicals, which has been used for the treatment of hyperlipidemia [6], nonproliferative diabetic retinopathy [7], and vitreous hemorrhage [8]. Previous reports have shown that active ingredients of PT can inhibit the apoptosis [9], improve insulin resistance [10], and increase insulin sensitivity [11]. PT is an effective TCM for the treatment of DR. However, due to the complexity of the active ingredients of PT, its molecular mechanism has not been elucidated clearly. Therefore, the objective of this study is to elucidate the active ingredients of PT correlated to the molecular mechanism of PT on the targets of DR.

Network pharmacology, an emerged discipline of Bioinformatics, is increasingly applied in TCM research in recent years. This approach is in accordance with the holistic theory that TCM emphasizes the treatment of diseases with multiple components and multiple targets [12]. Consequently, systematic pharmacology has been recognized as a suitable method to achieve the interaction between the active ingredients and various targets and explore the underlying mechanisms against complicated disease [13, 14]. Molecular docking technology, a key-and-lock principle, is a theoretical simulation approach to investigate the binding capacity of protein receptors to small medicine molecules using computers to study the receptors' characteristics as well as the interaction between receptors and medicinal molecules. We have referenced the previously published article [15] to investigate the mechanisms of PT on DR.

**Figure 1:**
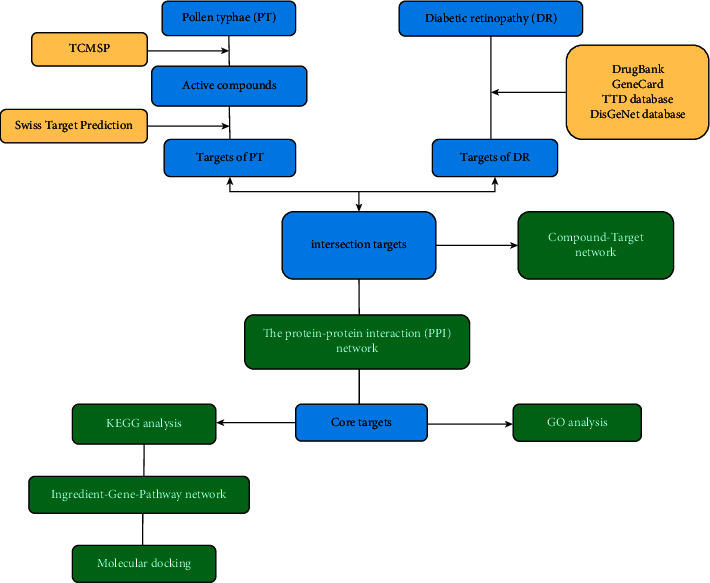
The workflow of this study.

In this study, the bioactive components of PT and the specific target proteins of PT and DR were combined to construct the pharmacological networks of “components-targets-diseases” and PPI. Moreover, several biologic pathways have been proposed to be involved in DR, for example, PI3K-Akt signaling pathways, VEGF signaling pathways, HIF-1 signaling pathways, and Rap1 and TNF signaling pathways. This suggests that it is of great significance to explore the characteristics of ingredients and targets of PT on DR in biological networks and molecular docking analysis. The workflow is summarized in Figure 1.

## 2. Materials and Methods

### 2.1. Collection of Chemical Ingredients and Related Targets in PT

The chemical ingredients of PT were gathered from TCMSP database (http://old.tcmsp-e.com/tcmsp.php). In order to select the ingredients, which have better pharmacokinetic properties and oral bioavailability in vivo, the ingredients were filtrated by suggested criterion in TCMSP database, and the ingredients whose drug-likeness (DL) ≥0.18 and oral bioavailability (OB) ≥30% were regarded as putative major ingredients and retained [16]. Additionally, the SMILES number of ingredients obtained from PubChem database was imported into the SwissTargetPrediction database through Probability^∗^ >0 to select the predicted targets.

### 2.2. Collection of the Targets of Diabetic Retinopathy

The DR-related targets were searched in the DrugBank (https://http://www.drugbank.ca/), GeneCards platform (https://www.genecards.org), Therapeutic Target Database (TTD), and DisGeNET database with “diabetic retinopathy” as the keywords [17, 18]. These collected targets were merged and duplicates were removed.

### 2.3. Establishment of Compound-Target Network and Analysis

To better demonstrate the anti-DR ingredients and targets of PT, Cytoscape 3.7.2 software was used to visualize the results and analyze the topological properties of the network. In the network, nodes represent ingredients or targets, and edges indicate compound-target gene interactions. In the topological analysis, the topological parameters were significant indexes to judge the importance of nodes, mainly including the degree of nodes and the betweenness centrality. Afterwards, the main active components were screened.

### 2.4. Protein-Protein Interaction Analysis

In order to explore the underlying mechanism of PT on DR, protein-protein interaction (PPI) networks were constructed using the STRING database and Cytoscape 3.7.2 software [19, 20]. The 171 intersection target genes were imported into the STRING database and the species were selected as “*Homo sapiens*” to obtain the interaction relationship between the targets, and it was saved in TSV format. Then, the file with TSV format was set into Cytoscape 3.7.2 software and the topology parameters were calculated to build an advanced protein-protein interaction network diagram and filter the core targets.

### 2.5. Gene Ontology and KEGG Pathway Enrichment Analysis

Gene ontology (GO) analysis and Kyoto Encyclopedia of Genes and Genomes (KEGG) are important methods to screen the key targets and enriched pathways in DAVID database (https://david.ncifcrf.gov/tools.jsp) setting *P* < 0.05 as the threshold [21, 22]. In this study, we chose the top 10 functional categories in biological process (BP), cellular component (CC), and molecular function (MF) and the top 20 DR-related KEGG terms, by using Bioinformatics (http://www.bioinformatics.com.cn/), to explore the key targets and the related pathways [23].

### 2.6. Construction of Ingredient-Gene-Pathway Network and Analysis

The ingredient-gene-pathway network was established by importing top 5 intersecting ingredients, 7 intersecting genes, and the leading 20 KEGG pathways into Cytoscape software. In this network, nodes represent ingredients, targets, and pathways, and edges indicate compound-target and target-pathway interactions. The topological parameters, mainly including the degree of nodes and the betweenness centrality, were utilized to evaluate the centrality features of nodes in ingredient-gene-pathway network.

### 2.7. Molecular Docking Analysis

Top five molecular compounds based on compound-target network were screened, and their molecular structures were downloaded from TCMSP. The top two key targets were selected by ingredient-gene-pathway network, and their PDB was obtained from Protein Data Bank (PDB) database. Then the PDB formats of proteins and the compounds were docked and visualized in the SYBYL-X 2.1.1 software, and the docking score was calculated to evaluate the binding degree between the bioactive components and the targets of PT. It is generally believed that the CSCORE >4.0 indicates that the docking molecules have good binding activity with the target.

## 3. Results

### 3.1. Screening of Active Ingredients and Related Targets

With the values of OB ≥30% and DL ≥0.18 properties, 8 active compounds, (2R)-5,7-dihydroxy-2-(4-hydroxyphenyl)chroman-4-one, arachidonic acid, isorhamnetin, quercetin, kaempferol, beta-sitosterol, testosterone palmitate, and kaempferol-3-O-*α*-L-rhamnosyl(1 ⟶ 2)-*β*-D-glucoside qt were selected from TCMSP database and 283 related targets of the compounds were screened from SwissTargetPrediction database. In addition, a total of 4077 DR-related targets were identified through the screening of the disease target database after removing the duplicate data. Then, the intersection of 171 targets was obtained.

### 3.2. The Compound-Target Network

**Figure 2:**
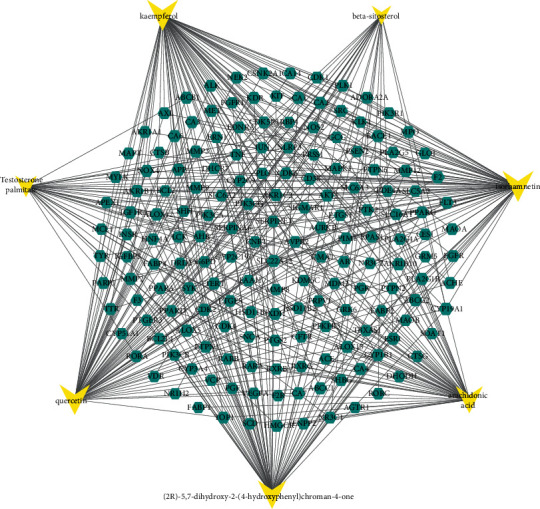
Active component-target network. The triangle node in yellow represents active component, and the hexagon node in green represents the intersection targets. The interaction between the components and the targets is represented by the edge.

In order to identify the interaction relationship between the ingredients and related targets, a compound-target network and topological analysis were made by using Cytoscape 3.7.2 and the degree values of compounds-targets were calculated. The higher the degree is, the closer the relationship between compounds and targets is, and the more important the compounds are in this network.  Figure 2 shows that five compounds probably were considered as significant therapeutic compounds in DR, namely, arachidonic acid, isorhamnetin, quercetin, kaempferol, and (2R)-5,7-dihydroxy-2-(4-hydroxyphenyl)chroman-4-one; and the seven ingredients are listed in Table 1.

### 3.3. Construction and Topological Analysis of the PPI Network

**Figure 3:**
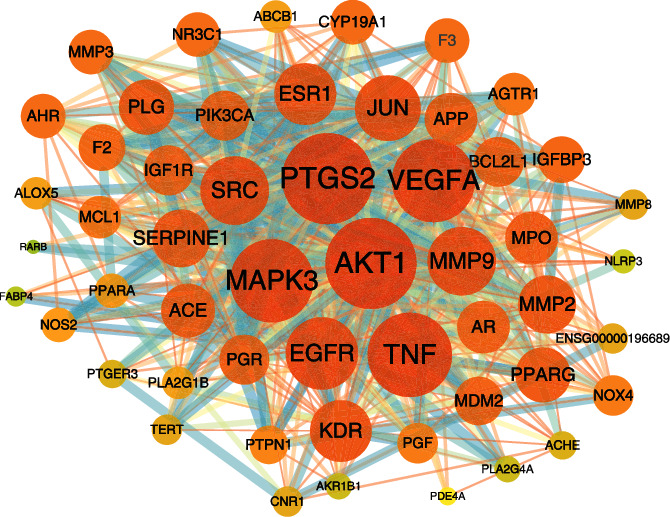
The PPI network. A darker color and larger node indicate a larger degree value, and the interaction between the components and the targets is represented by the edge.

The intersection targets PT and DR were inputted into the STRING database to obtain the PPI network, with 171 nodes and 1649 edges. Cytoscape 3.7.2 was used for merging and calculating topological analysis. The higher the degree is, the more important the targets are in this network. As is shown in Figure 3, the 53 key targets including the top six key targets (PTGS2, AKT1, VEGFA, MAPK3, TNF, and EGFR) were acquired, and the details of the 53 key targets are listed in Table 2. The PPI analysis demonstrated that these targets may be the key targets for PT at treating DR. In addition, the related abbreviations are provided in Supplementary Table S1.

### 3.4. GO Enrichment Analysis of Key Targets

**Figure 4:**
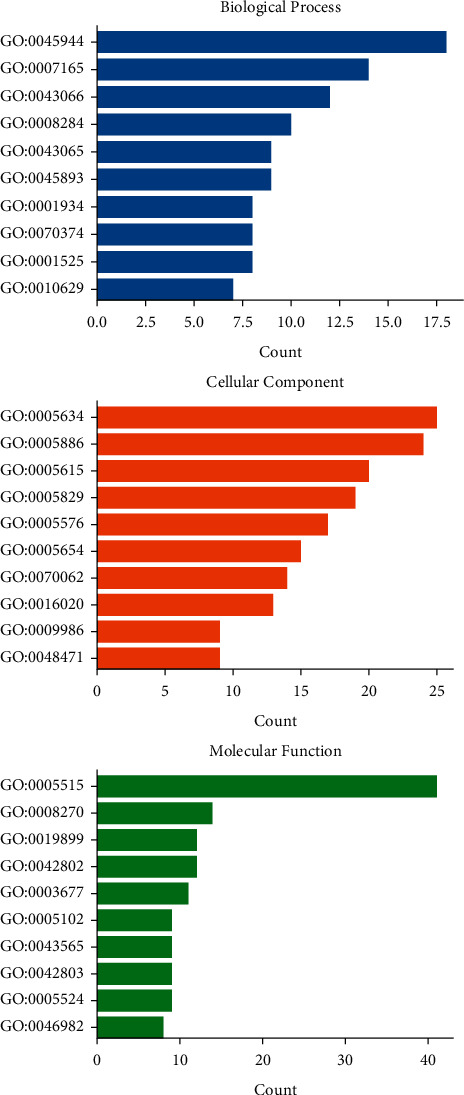
The diagram of the GO enrichment analysis. The *Y*-axis represents GO terms. The *X*-axis indicates the number of genes enriched.

GO enrichment analysis process consists of biological process (BP), cellular component (CC), and molecular function (MF). A total of GO terms, including 227 biological processes, 27 cellular components, and 55 molecular functions with *P* < 0.05, were obtained through inputting the 53 key genes into the DAVID website. Among them, the top ten most significantly enriched GO terms, respectively, were input into Bioinformatics (http://www.bioinformatics.com.cn/) to obtain the GO analysis diagram. As is shown in Figure 4, these targets were mainly enriched in signal transduction, cell proliferation, and apoptosis in BP, including positive regulation of transcription from RNA polymerase II promoter (GO:0045944), signal transduction (GO:0007165), negative regulation of apoptotic process (GO:0043066), positive regulation of cell proliferation (GO:0008284), and positive regulation of apoptotic process (GO:0043065). In CC analysis, the cellular components of these key targets mainly were nucleus (GO:0005634), plasma membrane (GO:0005886), extracellular space (GO:0005615), cytosol (GO:0005829), and extracellular region (GO:0005576). The MF analysis indicates that these key targets are closely related to protein binding (GO:0005515). These analyses suggested that PT may modulate angiogenesis by regulating the proliferation and apoptosis of retinal vascular cells, as well as the binding of core targets to its receptor, to treat DR. The details of GO enrichment are provided in Supplementary Table S2.

### 3.5. KEGG Enrichment Analysis

**Figure 5:**
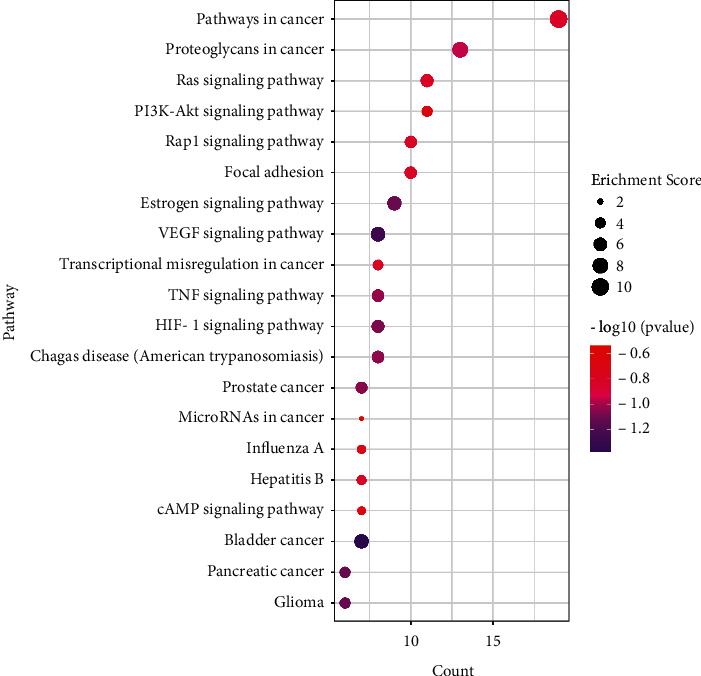
KEGG enrichment analysis. Count and pathway are represented by the *x*-axis and *y*-axis, respectively; the size and color of the dots indicate the enrichment score and the level of *P* value, respectively.

There were 80 KEGG signaling pathways obtained by the 53 key genes which were inputted into the DAVID database. Analysis of the KEGG pathway was carried out via Bioinformatics (http://www.bioinformatics.com.cn/), and a bubble diagram was obtained by listing the top 20 signaling pathways according to the enrichment score. As shown in Figure 5, the results of KEGG pathway enrichment included VEGF signaling pathway, PI3K-Akt signaling pathway, Rap1 signaling pathway, and HIF-1 signaling pathway, which were mainly related to oxidative stress, inflammation response, and retinal angiogenesis and proliferation. It is indicated that PT may act through these signaling pathways in treating PT. Furthermore, the top 20 signaling pathways and their specific related genes information are shown in Table 3.

### 3.6. The Ingredient-Gene-Pathway Network

**Figure 6:**
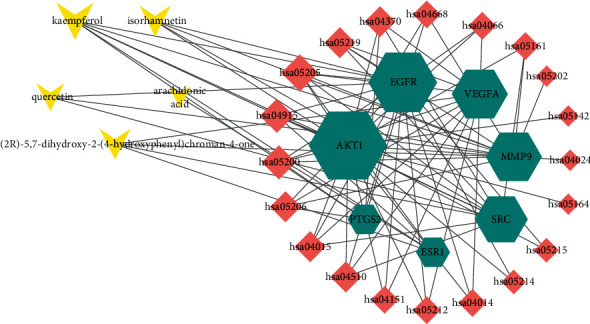
The ingredient-gene-pathway network. The triangle node in yellow represents active component, the hexagon node in green represents the intersecting targets, and the diamond node in red represents the signaling pathways. The interaction between the components and the targets is represented by the edge.

The ingredient-gene-pathway network consists of 29 nodes, including 5 active ingredients, 7 intersecting genes, and 20 pathways. As shown in Figure 6, AKTI, EGFR, and VEGFA are the core genes. According to the topological analysis, kaempferol and isorhamnetin are pivotal ingredients and could be the key effective components of PT for the treatment of DR. In this network, several pathways are involved in anti-DR, mainly including VEGF signaling pathway and Rap1 signaling pathway. It is indicated that PT may act through AKTI, EGFR, and VEGFA via VEGF signaling pathway and Rap1 signaling pathway in treating PT.

### 3.7. Molecular Docking Results

**Figure 7:**
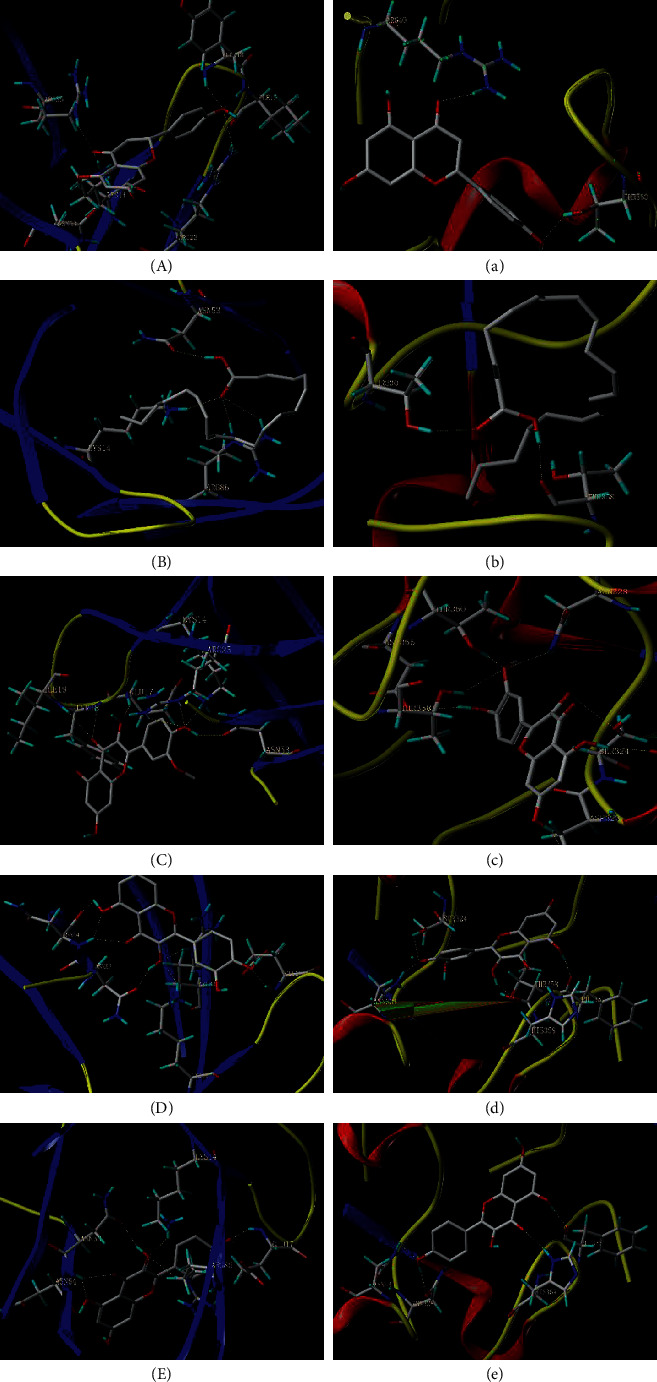
The model of molecular docking. (A) AKTI-(2R)-5,7-dihydroxy-2-(4-hydroxyphenyl)chroman-4-one; (B) AKTI-arachidonic acid; (C) AKT1-isorhamnetin; (D) AKT1-quercetin; (E) AKT1-kaempferol; (a) EGFR-(2R)-5,7-dihydroxy-2-(4-hydroxyphenyl)chroman-4-one; (b) EGFR-arachidonic acid; (c) EGFR-isorhamnetin; (d) EGFR-quercetin; (e) EGFR-kaempferol.

Five compounds had a docking score higher than 4.0 with EGFR and AKT1, suggesting good binding activity. All docking scores are enlisted in Table 4. As shown in Figure 7(A), (2R)-5,7-dihydroxy-2-(4-hydroxyphenyl)chroman-4-one stably bound to AKT1 through ARG23, ASN53, LYS14, ARG86, TYR18, and ILE19; arachidonic acid stably bound to the active site of AKT1 through hydrogen bonding interactions with ASN53, ARG86, and LYS14 on the AKT1 target protein (Figure 7(B)); isorhamnetin bound to the active site of AKT1 stably through hydrogen bond interactions with LYS14, ARG25, ASN53, GLU17, TYR18, and ILE19 on the AKT1 target protein (Figures 7(C)); quercetin and kaempferol stably bound to AKT1 through ASN54, ASN53, ARG86, GLU17, and LYS14 (Figures 7((D) and (E))); (2R)-5,7-dihydroxy-2-(4-hydroxyphenyl)chroman-4-one stably bound to the active site of EGFR through THR360 and ARG40 on EGFR target protein (Figure 7(a)); arachidonic acid stably bound to the active site of EGFR through hydrogen bonding interactions with THR358 and THR330 on the EGFR target protein (Figure 7(b)); isorhamnetin bound to EGFR stably through hydrogen bond interactions with ASP323, SER324, GLU320, THR358, ASP355, THR360, and ASN328 (Figure 7(c)); quercetin stably bound to EGFR through ASN331, SER324, THR358, HIS359, and PHE351 on the EGFR target protein (Figure 7(d)); kaempferol stably bound to the active site of AKT1 through PHE357, HIS359, ASN331, and ASN328 (Figure 7(e)).

## 4. Discussion

Diabetic retinopathy progresses through a variety of pathophysiological pathways, including the stimulation of VEGF in the eyes, oxidative stress, inflammation response, and the activation of the hexosamine pathway. The current treatments are monotherapy with limited efficacy, such as intravitreal injection of anti-VEGFs, laser and surgery, and other methods. Pollen Typhae (PT), a multicomponent and multitarget traditional Chinese medicine (TCM), has been clinically used in the treatment of DR and has a significant effect. The previous pharmacological research demonstrated that the components of PT have good antioxidant and anti-inflammatory effects [24]. Therefore, it is of great significance to further explore the active ingredients and molecular mechanism anti-DR. In our study, combining compound-target and ingredient-gene-pathway networks, we predicted that active constituents (arachidonic acid, isorhamnetin, quercetin, and kaempferol) are closely related to PT for DR. Based on the PPI analysis and ingredient-gene-pathway network, core target proteins (PTGS2, AKT1, VEGFA, MAPK3, TNF, and EGFR) were obtained. Considering the results of the KEGG pathway enrichment analysis and ingredient-gene-pathway network, the key signaling pathways (VEGF signaling pathway, PI3K-Akt signaling pathway, Rap1 signaling pathway, and HIF-1 signaling pathway) were selected. Arachidonic acid is one of the essential polyunsaturated fatty acids in human body and plays a key role in regulating inflammatory responses. Previous report indicated that 5,6-dihydroxytrienoic lactone, a stable metabolite of arachidonic acid, could mediate microvascular dilation to contribute to the anti-DR activity [25]. Furthermore, lots of experiments have shown that arachidonic acid and its metabolites can suppress inflammation [26] and reduce free radical generation to participate in the prevention of diabetes and its microvascular complications. Pollen Typhae is particularly rich in flavonoids, especially flavanol triglycosides including derivatives of isorhamnetin, quercetin, and kaempferol. Modern pharmacological studies have confirmed that flavonoids have good effect on the antioxidant and anti‐inflammation activities, regulation of blood glucose and lipids, and regulation of cell cycle and vascular integrity. Several studies have demonstrated that isorhamnetin has good effects on attenuation of diabetes-induced oxidative stress and protection of nerves [27, 28]. Studies have certified that quercetin can protect the nerves in diabetic rat retina and prevent diabetic retinopathy in rats by inducing heme oxygenase-1 expression [29, 30]. Researchers have found that quercetin has significant inhibitory activity against the expressions of MCP-1, MMP9, and VEGF and may have a promising role on the treatment of DR [31], and it also can relieve inflammatory responses by suppression of nuclear factor kappa B in mouse. In this study, ingredient-gene-pathway network shows that kaempferol is one of the core ingredients. Many experiments and findings have confirmed that kaempferol could protect retinal ganglion cells through regulating vasohibin-1 from high-glucose-induced injury [32] and prevent retinal pigment epithelial cell damage from oxidative stress [32]. Another study has indicated that kaempferol can inhibit VEGF and PGF expression and in vitro angiogenesis under high-glucose environment [33]. Therefore, these might demonstrate that the active ingredients of PT play a crucial role in developing microvascular events and it might be promising in the prevention and treatment of DR.

The PPI network analysis illustrates that gene targets (PTGS2, AKT1, VEGFA, MAPK3, and TNF) may be the main targets of PT to DR. The ingredient-gene-pathway network shows that AKT1 and EGFR are the core target genes. AKT1 is a suppressor gene and plays a key role in DR, and pharmacological studies have demonstrated that high glucose promotes retinal endothelial cell migration through activation of AKT1 [34, 35]. The previous studies have indicated that EGFR inhibitors played the important role in attenuating inflammatory infiltration and angiogenesis in mice with diabetic retinopathy [36]. Another in vivo study indicated that it can prevent pathological retinal neovascularization via inhibiting EP (4)R-EGFR-Gab1-AKT signaling pathway [37]. Studies and experiments have certified that regulating the EGFR pathway also could inhibit the proliferation, migration, and invasion of endothelial cells and retinal pericytes of diabetic retinopathy rats [38]. Lots of studies have suggested that PTGS2 could reduce the inflammatory response by downregulating the PTGS2 level [39, 40]. The study suggests that inhibition of p-c-Jun expression could prevent retinal neuronal cell death in NMDA-induced excitotoxicity [41]. Furthermore, inhibiting the activation of the stress response kinase JNK can improve insulin action in retina [42]. Jun was determined as a target of c-Jun N-terminal kinase and plays a critical role in regulating angiogenesis in vitro [43]. Jun was proved to be involved in improving hypoxia in retinal vascular endothelial cells by regulating cooperation of c-Jun/AP-1 and HIF-1alpha [44], which is potentially important for treatment of DR. Less relevant studies have shown that ESR1 has a direct effect on DR; however, some articles previously reported that ESR1 plays a critical role in glucose transporter GLUT4 regulation and maintains glycemic homeostasis [45, 46]. A previous research indicated that MAPK3 is a key target for treatment of rats with diabetic retinopathy [47].

Functional and enrichment analysis shows that these pathways (VEGF signaling pathway, PI3K-Akt signaling pathway, Rap1 signaling pathway, and HIF-1 signaling pathway) are significantly involved in antioxidative stress and anti-inflammation and suppress the angiogenesis of retina. Study suggested that modulating the PI3K/AKT signaling pathway could ameliorate diabetes-induced proliferative retinopathy [48, 49]. In vitro evidence showed that the activation of PI3K/Akt signaling pathway was closely related to oxidative stress inhibition and proinflammatory response of microglial cells associated with diabetic retinopathy [50]. Focal adhesion signaling pathway was important for maintaining the integrity of retinal vascular structure and function [51, 52]. According to the study results, those genes enriched in focal adhesion signaling pathway were considerably upregulated [53–55]. Moreover, proteomic bioinformatic analysis in DR indicated that DR development was closely related to focal adhesion and PI3K-Akt signaling pathway [56]. For the VEGF signaling pathway, one study's results suggested that the inhibition of the VEGF signaling pathway could protect diabetic retina, and this protective effect might be related to anti-inflammatory and antioxidative activities [57]. In vivo studies indicated that downregulation of the VEGF and HIF1 signaling pathway could inhibit the proliferation and angiogenesis of high-glucose-induced human retinal endothelial cells [58–60]. Additionally, lots of studies and experiments results have confirmed that downregulation of the VEGF and HIF1 signaling pathway could also prevent hypoxia-induced retinal angiogenesis [61–63]. One experiment demonstrated that HIF-1 signaling pathway had a protective effect on DR [64], and it is suggested that targeting the HIF-1 signaling pathway could be a therapeutic approach to prevent development of DR.

## 5. Conclusion

In summary, this study is aimed at illuminating the therapeutic effect on DR through network pharmacology and molecular docking analysis. We predicted that these active ingredients in PT, including arachidonic acid, isorhamnetin, quercetin, and kaempferol, and core targets, such as PTGS2, AKT1, VEGFA, MAPK3, TNF, and EGFR, mainly intervene in core targets through VEGF signaling pathway, PI3K-Akt signaling pathway, Rap1 signaling pathway, HIF-1 signaling pathway, and other signaling pathways, thereby exerting their roles in the treatment DR.

## Figures and Tables

**Table 1:** Information of 7 compounds in the pharmacological network.

Molecular ID	Molecular name	Molecular structure	OB (%)	DL
MOL001439	Arachidonic acid	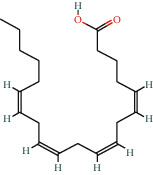	45.57	0.2

MOL000358	Beta-sitosterol	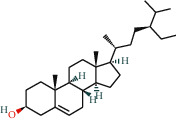	36.91	0.75

MOL000354	Isorhamnetin	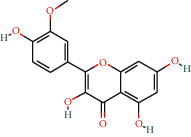	49.6	0.31

MOL000422	Kaempferol	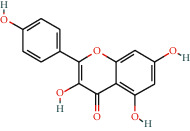	41.88	0.24

MOL000098	Quercetin	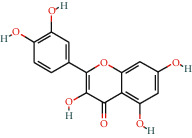	46.43	0.28

MOL006111	Testosterone palmitate	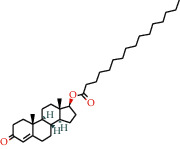	34.14	0.71

MOL001040	(2R)-5,7-Dihydroxy-2-(4-hydroxyphenyl)chroman-4-one	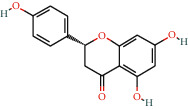	42.36	0.21

**Table 2:** The details of the 53 key targets.

Core targets	Protein name	UniProt ID
AKT1	RAC-alpha serine/threonine-protein kinase	P31749
VEGFA	Vascular endothelial growth factor A	P15692
MAPK3	Mitogen-activated protein kinase 3	P27361
TNF	Tumor necrosis factor	P01375
EGFR	Epidermal growth factor receptor	P00533
SRC	Proto-oncogene tyrosine-protein kinase Src	P12931
PTGS2	Prostaglandin G/H synthase 2	P35354
MMP9	Matrix metalloproteinase-9	P14780
JUN	Transcription factor AP-1	P05412
ESR1	Estrogen receptor	P03372
KDR	Vascular endothelial growth factor receptor 2	P35968
PIK3CA	Phosphatidylinositol 4,5-bisphosphate 3-kinase catalytic subunit alpha isoform	P42336
AR	Androgen receptor	P10275
MDM2	E3 ubiquitin-protein ligase Mdm2	Q00987
MMP2	72 kDa type IV collagenase	P08253
APP	Amyloid-beta precursor protein	P05067
PPARG	Peroxisome proliferator-activated receptor gamma	P37231
BCL2L1	Bcl-2-like protein 1	Q07817
ACE	Angiotensin-converting enzyme	P12821
IGF1R	Insulin-like growth factor 1 receptor	P08069
PGR	Progesterone receptor	P06401
SERPINE1	Plasminogen activator inhibitor 1	P05121
F2	Coagulation factor II	P00734
MPO	Myeloperoxidase	P05164
NR3C1	Nuclear receptor subfamily 3 group C member 1	P04150
MCL1	Induced myeloid leukemia cell differentiation protein Mcl-1	Q07820
PLG	Plasminogen	P00747
AGTR1	Type-1 angiotensin II receptor	P30556
CYP19A1	Cytochrome P450 19A1	P11511
IGFBP3	Insulin-like growth factor-binding protein 3	P17936
AHR	Aryl hydrocarbon receptor	P35869
PGF	Placenta growth factor	P49763
ABCB1	ATP-dependent translocase ABCB1	P08183
PTPN1	Tyrosine-protein phosphatase nonreceptor type 1	P18031
PPARA	Peroxisome proliferator-activated receptor alpha	Q07869
ACHE	Acetylcholinesterase	P22303
PLA2G4A	Cytosolic phospholipase A2	P47712
MMP3	Stromelysin-1	P08254
PLA2G1B	Phospholipase A2	P04054
ALOX5	Polyunsaturated fatty acid 5-lipoxygenase	P09917
TERT	Telomerase reverse transcriptase	O14746
F3	Coagulation factor III	P13726
CNR1	Cannabinoid receptor 1	P21554
NOX4	NADPH oxidase 4	Q9NPH5
MMP8	Matrix metalloproteinase-8	P22894
NOS2	Nitric oxide synthase	P35228
AKR1B1	Aldo-keto reductase family 1 member B1	P15121
PTGER3	Prostaglandin E2 receptor EP3 subtype	P43115
ENSG00000196689	none	none
FABP4	Fatty acid-binding protein 4	P15090
NLRP3	NACHT, LRR, and PYD domains-containing protein 3	Q96P20
PDE4A	cAMP-specific 3′,5′-cyclic phosphodiesterase 4A	P27815
RARB	Retinoic acid receptor beta	P10826

**Table 3:** The top 20 signaling pathways with related genes.

Term	Pathway	Genes	*P* value
hsa05200	Pathways in cancer	JUN, NOS2, MMP2, PTGER3, PTGS2, MMP9, EGFR, PGF, IGF1R, VEGFA, AR, PIK3CA, MDM2, AGTR1, AKT1, RARB, PPARG, BCL2L1, MAPK3	4.30*E* − 11
hsa05205	Proteoglycans in cancer	SRC, MMP2, ESR1, TNF, MMP9, EGFR, IGF1R, VEGFA, PIK3CA, MDM2, KDR, AKT1, MAPK3	7.30*E* − 09
hsa04014	Ras signaling pathway	PIK3CA, PLA2G1B, KDR, PLA2G4A, AKT1, EGFR, PGF, BCL2L1, IGF1R, MAPK3, VEGFA	2.62*E* − 06
hsa04151	PI3K-Akt signaling pathway	PIK3CA, MDM2, KDR, AKT1, EGFR, PGF, BCL2L1, IGF1R, MCL1, MAPK3, VEGFA	1.05*E* − 04
hsa04510	Focal adhesion	JUN, PIK3CA, SRC, KDR, AKT1, EGFR, PGF, IGF1R, MAPK3, VEGFA	9.89*E* − 06
hsa04015	Rap1 signaling pathway	PIK3CA, CNR1, SRC, KDR, AKT1, EGFR, PGF, IGF1R, MAPK3, VEGFA	1.16*E* − 05
hsa04915	Estrogen signaling pathway	JUN, PIK3CA, SRC, MMP2, AKT1, ESR1, MMP9, EGFR, MAPK3	3.25*E* − 07
hsa04370	VEGF signaling pathway	PIK3CA, SRC, KDR, PLA2G4A, AKT1, PTGS2, MAPK3, VEGFA	1.68*E* − 07
hsa04066	HIF-1 signaling pathway	PIK3CA, NOS2, SERPINE1, AKT1, EGFR, IGF1R, MAPK3, VEGFA	3.82*E* − 06
hsa05142	Chagas disease (American trypanosomiasis)	JUN, ACE, PIK3CA, NOS2, SERPINE1, AKT1, TNF, MAPK3	6.52*E* − 06
hsa04668	TNF signaling pathway	JUN, PIK3CA, MMP3, AKT1, PTGS2, TNF, MMP9, MAPK3	7.88*E* − 06
hsa05202	Transcriptional misregulation in cancer	IGFBP3, MMP3, MDM2, PPARG, MPO, MMP9, BCL2L1, IGF1R	1.39*E* − 04
hsa05219	Bladder cancer	SRC, MMP2, MDM2, MMP9, EGFR, MAPK3, VEGFA	3.13*E* − 07
hsa05215	Prostate cancer	AR, PIK3CA, MDM2, AKT1, EGFR, IGF1R, MAPK3	2.94*E* − 05
hsa05161	Hepatitis B	JUN, PIK3CA, SRC, AKT1, TNF, MMP9, MAPK3	4.68*E* − 04
hsa05164	Influenza A	JUN, PIK3CA, NLRP3, AKT1, PLG, TNF, MAPK3	0.001220965
hsa04024	cAMP signaling pathway	JUN, PIK3CA, PTGER3, PDE4A, AKT1, PPARA, MAPK3	0.002361912
hsa05206	MicroRNAs in cancer	ABCB1, MDM2, PTGS2, MMP9, EGFR, MCL1, VEGFA	0.013841455
hsa05212	Pancreatic cancer	PIK3CA, AKT1, EGFR, BCL2L1, MAPK3, VEGFA	8.07*E* − 05
hsa05214	Glioma	PIK3CA, MDM2, AKT1, EGFR, IGF1R, MAPK3	8.07*E* − 05

**Table 4:** Docking scores of active compounds of PT with core targets.

Targets	PDB ID	Compound	Molecular ID	CSCORE
AKT1	1unq	(2R)-5,7-Dihydroxy-2-(4-hydroxyphenyl)chroman-4-one	MOL001040	4
AKT1	1unq	Arachidonic acid	MOL001439	5
AKT1	1unq	Isorhamnetin	MOL000354	5
AKT1	1unq	Quercetin	MOL000098	4
AKT1	1unq	Kaempferol	MOL000422	4
EGFR	5w7b	(2R)-5,7-Dihydroxy-2-(4-hydroxyphenyl)chroman-4-one	MOL001040	4
EGFR	5w7b	Arachidonic acid	MOL001439	4
EGFR	5w7b	Isorhamnetin	MOL000354	4
EGFR	5w7b	Quercetin	MOL000098	3
EGFR	5w7b	Kaempferol	MOL000422	3

## Data Availability

The datasets in this study can be obtained from the corresponding author upon reasonable request. The authors are responsible for providing the final supplementary material files that will be published along with the article.

## Supplementary Materials

Supplementary Materials Table S1: the abbreviations. Table S2: the GO enrichment with related genes.
