# Supplementary material for: Investigating the Mechanisms of Pollen Typhae in the Treatment of Diabetic Retinopathy Based on Network Pharmacology and Molecular Docking
Source: Evid Based Complement Alternat Med. 2022 Jan 3;2022:5728408. doi: 10.1155/2022/5728408 (PMC8747905; doi:10.1155/2022/5728408)
Supplement: Supplementary Materials — Table S1: the abbreviations. Table S2: the GO enrichment with related genes. [file 5728408.f1.docx]

Table S1 The abbreviations.

| AKT1 | Serine/Threonine-protein Kinase 1 |
| --- | --- |
| BP | Biological Process |
| CC | Cellular Component |
| DAVID | The Database for Annotation, Visualization, and Integrated Discovery |
| DL | Drug-Likeness |
| DR | Diabetic Retinopathy |
| EGFR | Epidermal Growth Factor Receptor |
| ESR1 | Estrogen Receptor 1 |
| GO | Genome Ontology |
| HIF-1 | Hypoxia Inducible Factor-1 |
| KEGG | Kyoto Encyclopedia of Genes and Genomes pathway |
| MAPK3 | Mitogen-Activated Protein Kinase 3 |
| MF | Molecular Function |
| MMP9 | Matrix Metallopeptidase 9 |
| PI3K-Akt | Phosphoinositide-3-Kinase/Protein Kinase |
| PDB | Protein Data Bank |
| PPI | The Protein-Protein Interaction network |
| PT | Pollen Typhae |
| PTGS2 | Prostaglandin G/H Synthase 2 |
| SRC | Spare Respiratory Capacity |
| T2DM | Type 2 Diabetes Mellitus |
| TCM | Traditional Chinese Medicine |
| TCMSP | Traditional Chinese Medicine Systems Pharmacology |
| TNF | Tumor Necrosis Factor |
| TTD | Therapeutic Target Database |
| VEGF | Vascular Endothelial Growth Factor |
| VEGFA | Vascular Endothelial Growth Factor A |

Table S2

The GO enrichment with related genes.

| GO | Term | Genes | *P* value | Category |
| --- | --- | --- | --- | --- |
| GO:0045944 | positive regulation of transcription from RNA polymerase II promoter | APP, JUN, PLA2G1B, SERPINE1, AHR, NR3C1, ESR1, TNF, EGFR, VEGFA, AR, AKT1, NLRP3, RARB, PGR, PPARG, PPARA, MAPK3 | 2.10E-09 | Biological process |
| GO:0007165 | signal transduction | PLA2G1B, SRC, NR3C1, ESR1, EGFR, PGF, IGF1R, AR, AKT1, PDE4A, NLRP3, RARB, PGR, PPARG | 3.09E-05 | Biological process |
| GO:0043066 | negative regulation of apoptotic process | SRC, MDM2, KDR, RARB, AKT1, MPO, MMP9, EGFR, BCL2L1, IGF1R, MCL1, VEGFA | 9.18E-08 | Biological process |
| GO:0008284 | positive regulation of cell proliferation | AR, MDM2, KDR, RARB, F2, EGFR, PGF, BCL2L1, IGF1R, VEGFA | 9.76E-06 | Biological process |
| GO:0043065 | positive regulation of apoptotic process | CNR1, SRC, IGFBP3, RARB, NOX4, AKT1, PTGS2, TNF, BCL2L1 | 3.09E-06 | Biological process |
| GO:0045893 | positive regulation of transcription, DNA-templated | AR, JUN, SRC, PPARG, AHR, PPARA, ESR1, TNF, MAPK3 | 1.48E-04 | Biological process |
| GO:0001934 | positive regulation of protein phosphorylation | KDR, AKT1, F2, TNF, MMP9, EGFR, MAPK3, VEGFA | 1.05E-07 | Biological process |
| GO:0070374 | positive regulation of ERK1 and ERK2 cascade | JUN, SRC, KDR, NOX4, TNF, EGFR, MAPK3, VEGFA | 9.31E-07 | Biological process |
| GO:0001525 | angiogenesis | JUN, PIK3CA, MMP2, SERPINE1, KDR, PTGS2, PGF, VEGFA | 4.67E-06 | Biological process |
| GO:0010629 | negative regulation of gene expression | ACE, TERT, NOS2, AKT1, PGR, ESR1, TNF | 3.52E-06 | Biological process |
| GO:0005634 | nucleus | ACHE, SRC, AHR, NR3C1, PTGS2, MPO, EGFR, TERT, KDR, AKT1, NLRP3, MCL1, MAPK3, JUN, NOS2, MMP2, IGFBP3, ESR1, AR, FABP4, MDM2, RARB, PPARG, PGR, PPARA | 0.006604668 | Cellular component |
| GO:0005886 | plasma membrane | PTPN1, ACHE, APP, ACE, ABCB1, SRC, MMP2, PTGER3, SERPINE1, PLG, F2, F3, ESR1, TNF, EGFR, IGF1R, AR, TERT, PIK3CA, CNR1, MDM2, KDR, AGTR1, AKT1 | 3.10E-04 | Cellular component |
| GO:0005615 | extracellular space | ACHE, APP, ACE, PLA2G1B, MMP2, IGFBP3, MMP3, SERPINE1, AKR1B1, PLG, F2, MMP8, MPO, F3, TNF, MMP9, EGFR, PGF, VEGFA, ALOX5 | 1.39E-09 | Cellular component |
| GO:0005829 | cytosol | PTPN1, APP, JUN, NOS2, SRC, AKR1B1, PLA2G4A, AR, FABP4, PIK3CA, ALOX5, MDM2, AKT1, PDE4A, NLRP3, PPARG, MCL1, BCL2L1, MAPK3 | 0.002754926 | Cellular component |
| GO:0005576 | extracellular region | ACHE, APP, ACE, PLA2G1B, MMP2, IGFBP3, MMP3, SERPINE1, PLG, F2, MMP8, TNF, MMP9, PGF, VEGFA, KDR, NLRP3 | 4.62E-06 | Cellular component |
| GO:0005654 | nucleoplasm | JUN, AKR1B1, AHR, NR3C1, ESR1, AR, TERT, MDM2, AKT1, RARB, PGR, PPARG, PPARA, MCL1, MAPK3 | 0.018086641 | Cellular component |
| GO:0070062 | extracellular exosome | APP, ACE, ABCB1, SRC, IGFBP3, SERPINE1, AKR1B1, PLG, F2, MPO, F3, MMP9, FABP4, MAPK3 | 0.042385725 | Cellular component |
| GO:0016020 | membrane | ACHE, ACE, ABCB1, ESR1, CYP19A1, TNF, EGFR, PGF, IGF1R, VEGFA, PDE4A, MCL1, BCL2L1 | 0.016373507 | Cellular component |
| GO:0009986 | cell surface | ACHE, APP, ABCB1, PLA2G1B, PLG, TNF, F3, EGFR, VEGFA | 1.20E-04 | Cellular component |
| GO:0048471 | perinuclear region of cytoplasm | ACHE, APP, NOS2, SRC, PDE4A, AKR1B1, NOX4, PPARG, EGFR | 3.03E-04 | Cellular component |
| GO:0005515 | protein binding | APP, ACHE, ABCB1, SRC, SERPINE1, PLG, AHR, NR3C1, PTGS2, TNF, EGFR, IGF1R, TERT, ALOX5, KDR, AKT1, PDE4A, NLRP3, MCL1, MAPK3, PTPN1, JUN, NOS2, MMP2, IGFBP3, MMP3, F2, MMP9, F3, ESR1, PGF, VEGFA, AR, PIK3CA, MDM2, AGTR1, NOX4, PPARG, PGR, PPARA, BCL2L1 | 9.00E-05 | Molecular function |
| GO:0008270 | zinc ion binding | PTPN1, ACE, MMP2, MMP3, NR3C1, MMP8, ESR1, MMP9, AR, MDM2, RARB, PGR, PPARG, PPARA | 3.15E-05 | Molecular function |
| GO:0019899 | enzyme binding | PTPN1, APP, AR, JUN, SRC, MDM2, AKT1, PGR, PPARG, PTGS2, ESR1, EGFR | 3.52E-09 | Molecular function |
| GO:0042802 | identical protein binding | APP, JUN, MDM2, AKT1, PPARG, ESR1, TNF, MMP9, EGFR, BCL2L1, IGF1R, VEGFA | 1.14E-05 | Molecular function |
| GO:0003677 | DNA binding | APP, AR, JUN, TERT, RARB, PGR, PPARG, AHR, PPARA, NR3C1, ESR1 | 0.026281294 | Molecular function |
| GO:0005102 | receptor binding | APP, AR, NOS2, PLA2G1B, SRC, SERPINE1, PGR, PLG, F2 | 9.81E-06 | Molecular function |
| GO:0043565 | sequence-specific DNA binding | AR, JUN, NLRP3, RARB, PGR, PPARG, PPARA, NR3C1, ESR1 | 1.48E-04 | Molecular function |
| GO:0042803 | protein homodimerization activity | ACHE, JUN, TERT, NOS2, PTGS2, PGF, BCL2L1, MCL1, VEGFA | 0.001441414 | Molecular function |
| GO:0005524 | ATP binding | ABCB1, PIK3CA, SRC, KDR, NLRP3, AKT1, EGFR, IGF1R, MAPK3 | 0.078043665 | Molecular function |
| GO:0046982 | protein heterodimerization activity | JUN, AGTR1, AHR, EGFR, PGF, BCL2L1, MCL1, VEGFA | 4.67E-04 | Molecular function |
